# A Rare Case of Atypical Choroid Plexus Papilloma in an Adult Male Patient: A Case Report

**DOI:** 10.1155/crip/8927598

**Published:** 2025-04-11

**Authors:** Abebe Melis Nisiro, Teketel Tadesse Geremew

**Affiliations:** Pathology Department, College of Medicine and Health Science, Hawassa University, Hawassa, Ethiopia

**Keywords:** brain tumor, case report, choroid plexus carcinoma, choroid plexus papilloma, choroid plexus tumors

## Abstract

Choroid plexus tumors (CPTs) are rare neoplasms. Patient presentation varies depending on the location of the lesions. Gross total resection of primary lesions remains the gold standard for surgical treatment of CPTs. Here, we present the case of a 22-year-old male patient with 2-day history of abnormal body movement and headache who was found to have an enhancing mass of the lateral ventricle. The patient underwent craniotomy for gross-total resection of the lesion, with final histopathology demonstrating WHO Grade II aCPP.

## 1. Introduction

Choroid plexus tumors (CPTs) are papillary neoplasms derived from the choroid plexus epithelium and are typically located in the ventricles [1]. Choroid plexus papillomas (CPPs) are usually found in the lateral ventricle in children, and they are usually found in the fourth ventricle in adults [2]. Seventy-four percent of cases were found in the first 10 years of life, and 45% in the first year of life [2]. Though they only make up 0.3%–0.8% of total brain tumors, these tumors are more common in younger people, making up 2%–4% of brain tumors in children under the age of 15 and 10%–20% of those in infants under the age of 1 year [3]. CPTs are classified into three types by the World Health Organization (WHO): choroid plexus carcinoma (CPC) (malignant; WHO Grade III), CPP (WHO Grade II; with intermediate features), and benign and more differentiated CPP (WHO Grade I) [1, 4]. Atypical choroid plexus papilloma (aCPP) is defined as a CPP that has increased mitotic activity; however, it does not fulfill the criteria for malignancy (high cellularity, brisk mitotic activity, nuclear pleomorphism, blurring of the papillary growth pattern, necrosis, and diffuse brain invasion) of CPC [1, 4]. Tumors of the lateral ventricle can cause specific deficits including hemiparesis, papilledema that results in blindness, convulsions, and mental abnormalities [5, 6]. MRIs of the brain and spine (with and without gadolinium) are used to evaluate the extent of the disease in patients diagnosed with aCPP [7]. In all CPTs, complete surgical tumor resection is considered the mainstay of treatment as well as an important prognostic factor [1, 7, 8].

## 2. Case

A 22-year-old male patient presented with abnormal body movements of 2 days duration that occur frequently in a day and night; each episode lasts for 5–10 min with a postictal sleep state. Finally, the patient developed a loss of consciousness for 20 min, with associated high-grade persistent fever and shortness of breath. Otherwise, he has no history of trauma to the head or other sites.

On physical examination on general appearance, the patient looked acutely sick and in distress, having tachypnea, tachycardia, and febrile. On examination, there is clear media, pupils are slightly reactive, lens is transparent, fundus shows pink disc, and fine crepitation is noted over lower chest with GCS: 14/15.

For the clinical impression of an intracranial space-occupying lesion+aspiration pneumonia+status epilepticus, he was investigated with laboratory tests.

On complete blood count WBC: leukocytosis with neutrophil predominance, Hgb and platelet- normal. ESR-increased, RBS -normal, renal and liver function test- normal, serum electrolytes- normal, HBV and HCV-negative and PICT-nonreactive.

On CSF analysis, it was unremarkable. No gram stain or AFB stain showed bacilli.

Brain MRI scan showed that there is a 3.3 × 3 cm lobulated frond-like T1 isointense and T2 isointense intraventricular choroid plexus of the left ventricle with homogeneous enhancement. There is dilation of both lateral ventricles with third and fourth ventricles; the brain parenchyma is normal in signal intensity, with no evidence of mass, hematoma, or shift of midline structures, with the conclusion of the left lateral ventricle choroid plexus lobulated frond-like T1 isointense and T2 isointense with homogeneous enhancement and communicating hydrocephalus suggestive of CPP.

For this, he underwent craniotomy and gross total resection. In addition, he was put on broad-spectrum antibiotics and antiepileptic medications.

The pathology unit received specimens in 10% neutral buffered formalin.

On gross morphologic examination, there were multiple gray-white to gray-brown tissue fragments measuring 2 cm in aggregates.

**Figure 1:**
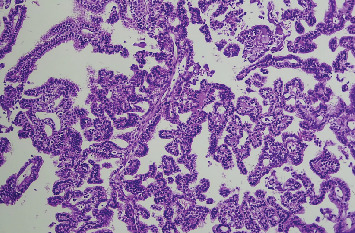
Low power view (4x) composed of branching finger-like papillary architecture with fibrovascular core lined by single to pseudostratified cuboidal to columnar epithelium (Figures 1, 2, 3, and 4).

**Figure 2:**
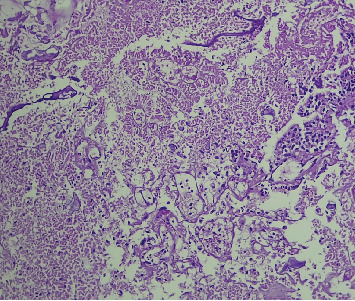
Mid power view (20x) shows the area of necrosis in the tumor.

**Figure 3:**
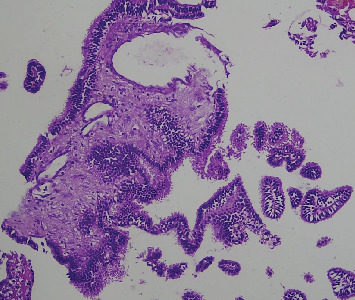
High power view (40x) composed of papillary architecture with fibrovascular core lined by pseudostratified cuboidal to columnar epithelium exhibiting moderate pleomorphism. A focal area of blurring of papillary architecture was evident.

**Figure 4:**
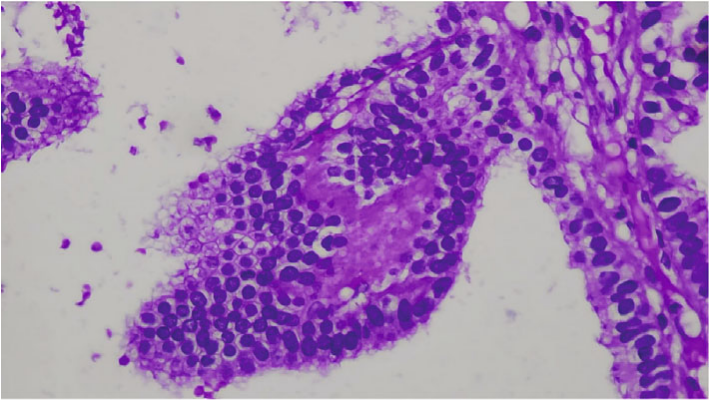
High power view (40x) composed of solid sheets of round to oval cells exhibiting moderate pleomorphism, hyperchromasia, and a high *N*/*C* ratio.

Microscopic examination shows tissue composed of branching finger-like papillary architecture with fibrovascular core lined by single to pseudostratified cuboidal to columnar epithelium exhibiting moderate pleomorphism. There are two mitotic figures/10 hpf, a geographic area of necrosis, frequent psammomatoid calcification, and a focal area of blurring of papillary architecture that was evident (Figures 1, 2, 3, and 4). S100 and CK were done, and they were positive.

Subsequently, follow-up CT and MRI scans were done, which show no residual mass left, and he is on follow-up schedule every 3 months with a good clinical condition.

## 3. Discussion

CPTs are a diverse group of neoplasms that range from well-differentiated papillomas (WHO Grade I) to very aggressive CPCs (WHO Grade III), with rare intermediate forms known as “aCPP” whose biologic behavior remains unknown [6, 9]. aCPP are rare intermediate types of CPT, characterized as a CPP with elevated mitotic activity but does not meet the criteria for CPC [1]. aCPP was first described in 1990 [10]. In 2007, the WHO added it to the central nervous system tumor classification and classified it as WHO Class II [10–12]. It most commonly affects youngsters, with the lateral ventricle accounting for 83%, followed by the third ventricle (13%), and the fourth ventricle (3%) (Table 1) [10, 13]. Unlike the typical age, our patient is an adult. CPP's clinical presentation may include signs and symptoms of obstructive hydrocephalus, vertigo, diplopia, lateral gaze palsies, and visual field abnormalities, but vary depending on the location [14, 15]. CPT of the lateral ventricle presentation include convulsions, mental abnormalities, papilledema leading to loss of eyesight, and localized deficits including hemiparesis [16]. Symptoms of this tumor in the fourth ventricle include headache, ataxia, nystagmus, cerebellar signs, dizziness, loss of vision, vomiting, and diplopia [17]. On CT and MRI scans, CPPs usually present as isodense or hyperdense, T1-isointense, T2-hyperintense, irregularly contrast-enhancing, well-delineated masses within the ventricles, but irregular tumor margins and disseminated disease may occur [2, 18]. MRI scans cannot distinguish it from the other two choroid plexus epithelial tumors, and the diagnosis is based on histology [10]. Intraoperative observations in rare aCPPs demonstrate a highly vascular tumor with a propensity to bleed, but this feature is also observed in CPPs [7].

Currently, histological diagnostic criteria recommended by the WHO is that mitotic count in CPP > 2 mitoses per 10 randomly selected high-power fields (with one high-power field corresponding to 0.23 mm^2^); besides, one or two of the following four features may also be present: increased cellularity, nuclear pleomorphism, blurring of the papillary pattern (solid growth), and areas of necrosis; however, these features are not required for a diagnosis of aCPP [10, 19]. Based on this criteria our case mitosis of 2/10 hpf, area of necrosis and solid sheet growth pattern so it is graded as aCPP. According to the morphological diagnostic criteria above, it can be distinguished from CPP and CPC [10].

Gross total resection of primary lesions and associated implants remains the gold standard for surgical treatment of CPP [1, 13, 20]. Our patient was managed with GTR. There are no prophylactic treatments for preventing CPP metastasis [13]. Histological appearance may not always indicate biologic behavior [11]. aCPP had a 5-year survival rate of 89%, which was between CPP and CPC [10]. Metastatic disease is even less common, more typically associated with CPC [21]. However, there is evidence that the diagnosis of atypical choroidal papilloma is associated with prognosis in children older than 3 years and adults but not in children younger than 3 years, who may have a favorable prognosis even with highly proliferative CPP [10].

## 4. Conclusion

aCPPs are rare intermediate forms of CPT, which most commonly occurs in youngsters. Despite the typical age of onset being young children, it can also occur in adults, like our patients. Therefore, physicians should be aware that CPTs can be a differential diagnosis for young adults with intracranial lesions. The definitive diagnosis is depending on the histopathology findings, and grading is important for prognostication purpose.

## Figures and Tables

**Table 1:** Similar cases from previous literatures.

**Case/series**	**Age, sex**	**Location**	**Presentation**	**Management**
Lee, S. H., Park, B. J., Kim, E. J., & Lim, Y. J. (2009). Atypical Choroid Plexus Papilloma in an Adult. *Journal of Korean Neurosurgical Society*, *46* (1), 74–76.	62, F	Lateral ventricle	Body weakness	Gross total resection
Goel, K., Birdi, U., Menaker, S., Bannykh, S. I., & Patel, C. (2022). Atypical Choroid Plexus Papilloma of the Fourth Ventricle in an Adult: A Case Report. *Cureus*, *14* (5), e25256.	47, male	Forth ventricle	Headache and seizure	Gross total resection
Menon, Ritu R et al. “Infratentorial Atypical Choroid Plexus Papilloma in an Adult: A Case Report and Literature Review.” *Indian journal of cancer*	41, F	Fourth ventricles	Headache and neck pain	Gross total resection
Jusué-Torres, Ignacio et al. “Papiloma atípico de los plexos coroideos en el adulto: publicación de un caso clínico y revisión de la bibliografía” [Atypical Choroid Plexus Papilloma in Adults: Case Report and Literature Review]. *Neurocirugia (Asturias, Spain)* vol. 23,3 (2012): 116-21.	38, male	Forth ventricle	Headache	Totally resected by posterior fossa craniectomy and telovellar approach

## Data Availability

The data used to support the findings of this study are available from the corresponding author upon request.
